# Electronic Raman scattering as an ultra-sensitive probe of strain effects in semiconductors

**DOI:** 10.1038/ncomms8136

**Published:** 2015-05-28

**Authors:** Brian Fluegel, Aleksej V. Mialitsin, Daniel A. Beaton, John L. Reno, Angelo Mascarenhas

**Affiliations:** 1 National Renewable Energy Laboratory, 15013 Denver West Parkway, Golden, Colorado 80401, USA; 2 Center for Integrated Nanotechnologies, Sandia National Laboratories, Albuquerque, New Mexico 87123, USA

## Abstract

Semiconductor strain engineering has become a critical feature of high-performance electronics because of the significant device performance enhancements that it enables. These improvements, which emerge from strain-induced modifications to the electronic band structure, necessitate new ultra-sensitive tools to probe the strain in semiconductors. Here, we demonstrate that minute amounts of strain in thin semiconductor epilayers can be measured using electronic Raman scattering. We applied this strain measurement technique to two different semiconductor alloy systems using coherently strained epitaxial thin films specifically designed to produce lattice-mismatch strains as small as 10^−4^. Comparing our strain sensitivity and signal strength in Al_*x*_Ga_1−*x*_As with those obtained using the industry-standard technique of phonon Raman scattering, we found that there was a sensitivity improvement of 200-fold and a signal enhancement of 4 × 10^3^, thus obviating key constraints in semiconductor strain metrology.

## Abstract (web summary)

Engineering strain in semiconductor structures provides additional control over the optical and electronic properties, which is promising for device applications. Fluegel *et al*. show that electronic Raman scattering provides a route to sensitively measure the degree of strain in thin semiconductor layers.

## Graphical abstract

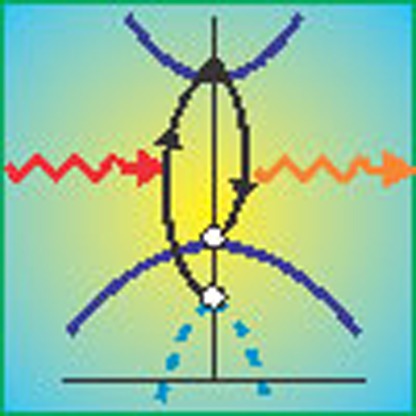

The effect of strain on the electronic, optical and vibrational properties of semiconductors is currently used for engineering advanced electronic and photonic devices. From nanometre-scale electronics with III–V compound semiconductors^1^ and strain scaling for CMOS to transforming silicon and germanium into suitable materials for photonic applications^2^,^3^, strain engineering has become a critical component in the design and manufacturing processes required to enable these advances. The most fundamental change that results from strain occurs to the semiconductor electronic band structure, which is conventionally probed using ellipsometry^4^,^5^, modulated reflectance^6^ and photoluminescence excitation spectroscopies^7^. Here, we report a new approach based on electronic Raman scattering^8^,^9^, which can measure the epilayer strain as low as 10^−4^ with orders-of-magnitude lower power and significantly higher sensitivity than vibrational Raman methods. The sensitivity of this approach rivals that of contemporary techniques and opens a new realm to optically probe effects of strain on electronic band structure.

Atomic displacements that result from the lattice strain in a semiconductor can be directly measured using various electron beam- or X-ray-based techniques. Amongst these, the dark-field electron holography technique^10^ can image the strain with high spatial resolution (2 nm) and high precision (2 × 10^−4^). X-ray techniques^11^ can probe the strain in thin semiconductor epilayers with high precision (10^−4^) but require synchrotron facilities to improve the spatial resolution^12^. The electron beam techniques involve extensive sample preparation, which is necessarily destructive and can modify the strain in the sample^10^. Furthermore, to accurately determine the strain, both of these approaches require complex simulations and a reference sample for comparison.

Raman scattering from phonons in semiconductors is an indirect technique for probing the strain^13^,^14^. This technique requires no sample preparation and is non-destructive^15^. With a spatial resolution better than 0.5 μm and a precision better than 10^−3^, this technique can be conveniently used to map the strain^16^ in semiconductor epilayers and large-area devices. Other spectroscopic techniques such as ellipsometry^4^,^5^, modulated reflectance^6^ and cathodoluminescence^17^ indirectly probe the strain through the changes it induces in the electronic band structure; however, they suffer from either lower spatial resolution or lower precision. In this work, we report an optical technique based on electronic Raman scattering to measure the effect of minute strain on the electronic band structure^8^. This technique is of unique value in investigating the heteroepitaxy of semiconductors, where understanding the role of strain and strain relaxation in metamorphic or pseudomorphic epilayers, which are required for multijunction solar cells^18^, diode lasers^3^ or photodetectors^2^, can be critical.

In electronic Raman scattering, the Raman shift of the incident laser probe is related to an electronic transition as opposed to a vibrational transition in conventional Raman scattering^8^,^13^,^14^. One example of this effect is the interband electronic Raman scattering process^19^,^20^,^21^, which is shown in Fig. 1a. A direct-gap, compressively strained semiconductor is illustrated for clarity, but the principle also holds for indirect transitions or tensile strain. Here, the Raman shift results from the transition of a hole from the heavy-hole band to the light-hole band^22^, whose separation in energy is induced by biaxial strain in a semiconductor with cubic symmetry^5^. Under resonance conditions, an electron that is excited from the light-hole band to the conduction band subsequently recombines with a hole in the heavy-hole band. This electronic Raman scattering process involves intervalence-band scattering of intrinsic photogenerated holes via the conduction band as an intermediate state^8^,^9^,^22^. Here, we demonstrate that this inelastic light-scattering process is an ultra-sensitive probe that can be used to measure the values of sufficiently small strain to determine the lattice mismatch between thin Al_*x*_Ga_1−*x*_As epilayers and the GaAs substrate on which they are grown when the Al concentration is as low as 6.7%.

## Results

### Strain measurement in nearly lattice-matched Al_*x*_Ga_1−*x*_As

**Figure 1:**
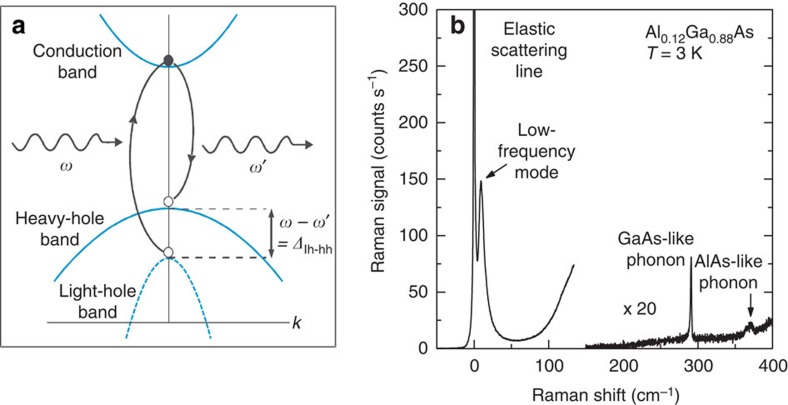
Raman spectrum overview. (**a**) Schematic of the intrinsic intervalence-band electronic Raman scattering: the Al_*x*_Ga_1−*x*_As electronic bands that participate in the three-band Raman scattering process are shown. Wavy lines denote the incoming (*ω*) and outgoing (*ω*′) photons. The thin solid lines denote the scattering of a hole from the heavy-hole band to the light-hole band via the generation of an electron in the conduction band intermediate state. The Raman shift of the observed low-frequency mode results from the biaxial compressive strain-induced splitting energy Δ_*lh-hh*_. (**b**) The Raman scattering spectrum from the AlGaAs epilayer with 12% Al content grown on a GaAs substrate is separately shown for the low-frequency (electronic Raman scattering) and high-frequency (phonon Raman scattering) regions. The low-frequency spectral window, which was recorded using laser excitation at 1.668 eV and 12 μW of power in the depolarized scattering geometry, shows a low-energy electronic Raman scattering feature near the elastically scattered laser line. The high-frequency region, which was recorded using laser excitation at 1.745 eV and 2 mW of power in the polarized scattering geometry, shows the GaAs-like phonon at 291 cm^−1^ and the AlAs-like phonon at 370 cm^−1^.

**Figure 2:**
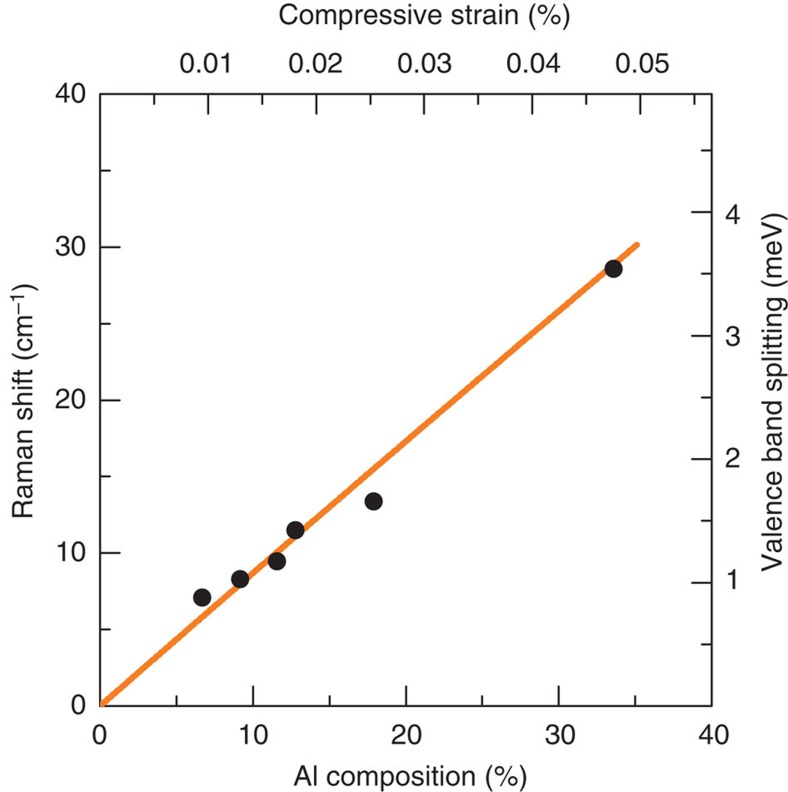
Energy of the low-frequency mode versus the theoretically predicted strain-induced valence-band splitting. The solid line displays the splitting between the light-hole and heavy-hole valence bands (see Fig. 1a) resulting from the lattice mismatch between the AlGaAs epilayer on the GaAs substrate, which was calculated using elastic moduli values at 0 K. The symbols show the energy of the low-frequency mode averaged over the dispersion for the resonance profile (see Fig. 3).

A set of thin Al_*x*_Ga_1−*x*_As epilayers that were grown on (001) GaAs substrates was studied using the aforementioned technique. In Fig. 1b, which shows the Raman spectrum for a sample with 12% Al, the expected vibrational Raman scattering peak from the GaAs-like longitudinal optic phonon mode is observed immediately below 300 cm^−1^. A new feature is observed in close proximity to the laser line; the light-scattering cross-section of this feature is substantially greater than that of the phonon. The position of this low-frequency mode is observed to correlate with the Al content in all other studied samples, as shown in Fig. 2. The energy of this mode correlates with the splitting of the heavy- and light-hole bands that results from the biaxial compressive strain because of lattice mismatch between the thin Al_*x*_Ga_1−*x*_As epilayer and the GaAs substrate (see the Methods section). The slope of the data in Fig. 2 shows that the scattering frequency is much more precise to the strain differentials than is the phonon frequency. The rate of change of the frequency of this mode is 600 cm^−1^per percent of the biaxial mismatch strain, which is more than two orders of magnitude greater than the measured 3 cm^−1^per percent strain-induced frequency shift^23^ for the GaAs longitudinal optic phonon as a function of the surface stress.

**Figure 3:**
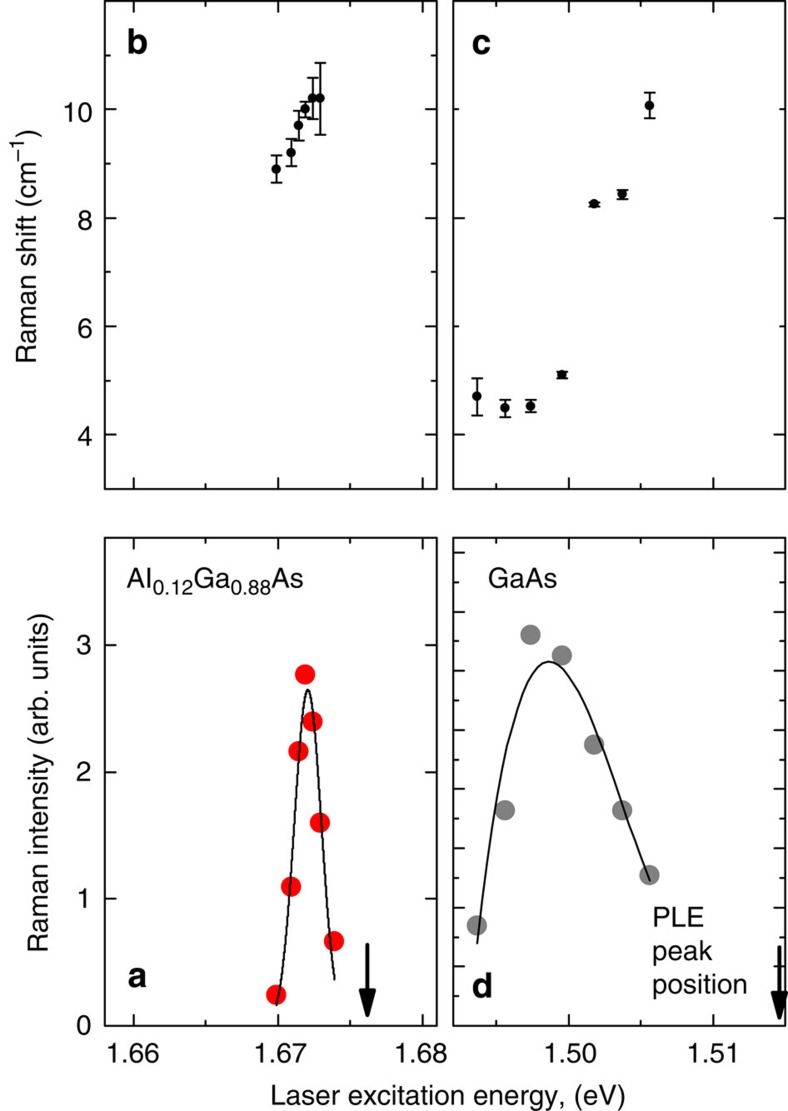
Resonance Raman profile and frequency dispersion of the low-frequency mode. (**a**) Resonance Raman profile of the low-frequency mode for 12% Al_*x*_Ga_1−*x*_As. (**b**) Frequency dispersion of the resonance Raman peaks that were measured in **a**. (**c**,**d**) Frequency dispersion and excitation profile of the low-frequency mode in GaAs. Error bars are the uncertainty in subtracting the underlying photoluminescence similar to that shown in Fig. 1b. The solid lines are smooth fits to the data. The arrows denote the maximum of the PLE peak that corresponds to the band edge of the epilayer.

A resonance Raman^7^,^24^ scattering study provided evidence that the low-energy feature originates from interband electronic Raman scattering. When the frequency of the laser excitation approaches the bandgap of the Al_*x*_Ga_1−*x*_As alloy, the intensity of the low-energy feature dramatically increases. The resonance profile is shown in Fig. 3a for a sample with a 12% Al content. The resonance is sharp, with an FWHM of 2.45 meV. Photoluminescence excitation spectroscopy (PLE) measurements that were performed to determine the exciton absorption edge indicate that the peak of the intensity resonance occurs only 2.9 meV below the PLE peak. The frequency dispersion of the low-energy feature as it undergoes resonance is shown in Fig. 3b. With respect to the laser energy, its slope ranges from flat to a few × 10^−2^ for various Al concentrations that were studied. Positive dispersions in the much larger range of 4 × 10^−1^ have been observed in the interband electronic Raman scattering of unstrained p-doped GaAs and are explained by the diverging valence bands at large wavevectors^20^. In the present case, the much smaller Fermi energy of the residual doped or photogenerated holes limits the interband Raman to *k*≈0, which results in a flatter dispersion. In the absence of any zone-centre valence band splitting, that is, for GaAs in Fig. 3c,d, the only remaining light scattering occurs from transitions at *k*≠0. As in the work of Olego and Cardona^20^, these transitions have a broader resonance. This helps to distinguish them from the strain-induced signal.

### Tensile strain in the highly mismatched alloy GaAs_1−*x*_N_*x*_

**Figure 4:**
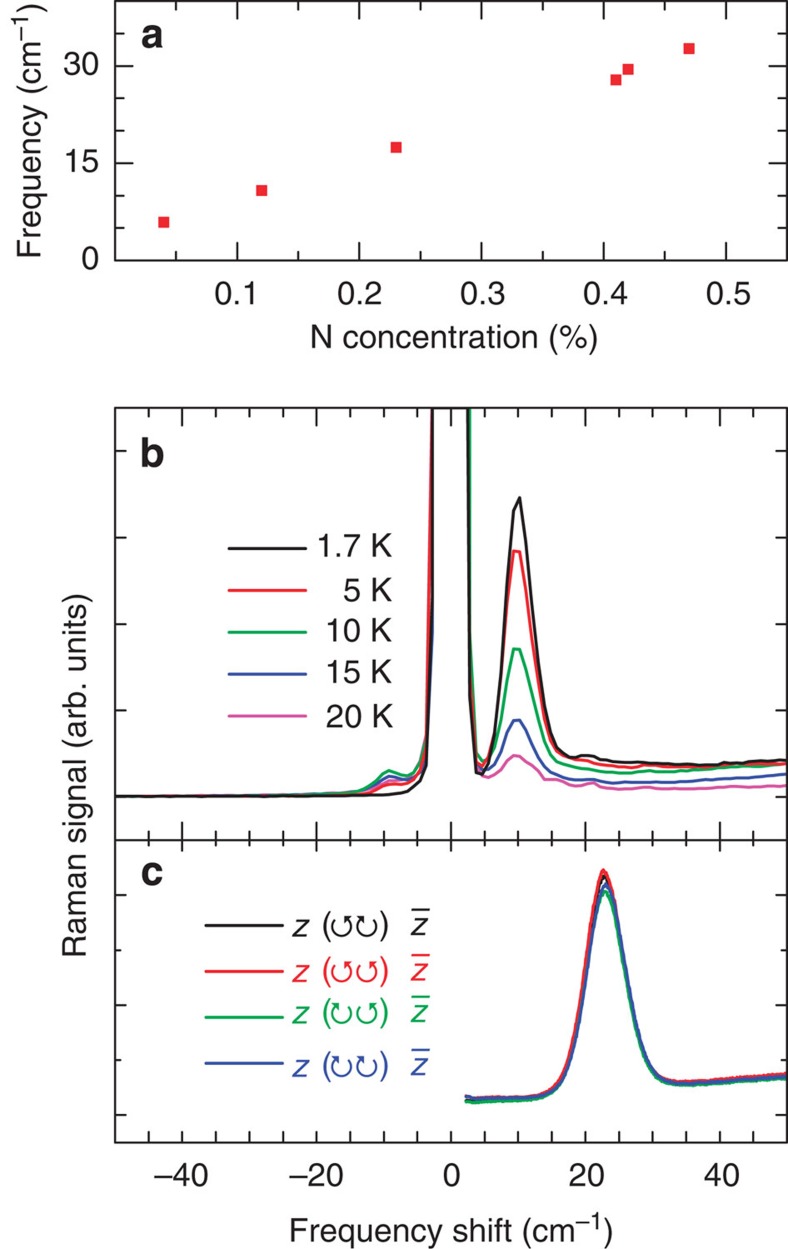
Electronic Raman scattering in GaAs_1−*x*_N_*x*_. (**a**) The 1.7-K electronic Raman scattering frequency for a set of GaAs_1−*x*_N_*x*_ samples with various N concentrations. The frequency was determined from spectra similar to **b**. (**b**) Stokes and anti-Stokes scattering components in a 300-nm-thick epilayer of GaAs_0.9988_N_0.0012_, which was strained to the GaAs substrate, were measured at various lattice temperatures. The 16-μW laser excitation is at 1.470 eV, which is below the GaAs_1−*x*_N_*x*_ bandgap energy. (**c**) Polarization analysis of the Raman scattering in a sample with *x*=0.0032 at 3.0 K. The circular labels in the Raman notation identify the excitation and collection polarizations as either left or right circularly polarized, and 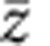 is the growth direction.

Light-scattering strain measurements are not limited to the Al_*x*_Ga_1−*x*_As system. GaAs_1−*x*_N_*x*_ is a highly size-mismatched mixed-anion alloy in which minute concentrations of N cause notably strong changes, such as a giant bandgap bowing^25^, in the alloy's electronic properties. Despite these key differences between the GaAs_1−*x*_N_*x*_ and Al_*x*_Ga_1−*x*_As systems, the electronic Raman scattering technique can directly measure the inverted valence-band splitting induced by N concentrations as low as *x*=5 × 10^−4^, as shown in Fig. 4. As in the case of Al_*x*_Ga_1−*x*_As, the GaAs_1−*x*_N_*x*_ electronic mode's frequency increases with increasing strain, as plotted in Fig. 4a. The strong resonance also enables detailed observation of the polarization independence in Fig. 4c and the anti-Stokes scattering component in Fig. 4b. The latter is observed in the small peak at negative frequency shift. This shift corresponds to an increase of the photon energy, that is, the inverse of the process shown in Fig. 1a. This energy increase is fuelled by the thermal occupation of holes in the lower valence band and occurs at temperatures ≥5 K. At higher temperatures, both low-frequency features thermalize because the thermally induced transitions between the light- and heavy-hole bands wash out the coherence of the electronic Raman process. A similar temperature dependence was observed in the case of Al_*x*_Ga_1−*x*_As.

## Discussion

A key limitation in high-spatial-resolution Raman spectroscopy is that, when the laser probe size is decreased, the required power density to maintain reasonable Raman scattering intensities becomes sufficiently high to cause local heating effects. In this regard, as the ratios of signal strengths to laser powers in Fig. 1b indicates, the much lower required power (≈4 × 10^3^ lower power) for electronic Raman scattering compared with phonon Raman scattering can be significantly advantageous. In strongly resonant cases, as observed in Figs 1b and 4b, the signal can be concurrently measured with the laser, which negates the requirement for a Raman spectrometer or other laser filtering.

In addition, a comparative reference of unstrained material, which is required in other strain measurement techniques, is not necessary because the electronic Raman shift is a direct measure of strain. The measured strain at low temperatures can be mapped to the corresponding strain at room temperature using the temperature dependence of the lattice constants^26^; such low-temperature measurements are necessary in the case of small valence-band splittings.

The advent of silicon-based photonics^2^ and III–V-based nanoelectronics^1^ and the integration of photonics and electronics^27^ require new metrology tools^17^ to probe the effects of the design or process technology variation on the electronic band structure^28^. The ultra-sensitivity to strain-induced band-structure changes, which are demonstrated for the electronic Raman technique, can make this technique a valuable tool in such applications. It can also be a valuable tool for probing electronic-structure changes induced by single dislocations^29^, strain engineering of ultra-thin semiconductor crystalline layers^30^ and unconventional approaches to heteroepitaxy^31^.

## Methods

### Growth

The set of 0.4–0.5-μm-thick, undoped Al_*x*_Ga_1−*x*_As epilayers was grown using molecular beam epitaxy on commercial semi-insulating (001) GaAs substrates. GaAs_1−*x*_N_*x*_ epilayers with a thickness of 0.3 μm were also grown using molecular beam epitaxy on GaAs substrates. Because all epilayer thickness values were much lower than the critical thickness for the grown films, the films were assumed to be perfectly pseudomorphically strained to the substrate. The film compositions reported here were determined using PLE in the case of Al_*x*_Ga_1−*x*_As or X-ray diffraction in the case of GaAs_1−*x*_N_*x*_.

### Strain estimate

The accepted values for the lattice constants for GaAs (5.6525 Å) and AlAs (5.6605 Å) at 10 K were used to estimate the strain values in Fig. 2. The in-plane biaxial strain component is given by *ɛ*_‖_=−(*a*_AlGaAs_−*a*_GaAs_)/*a*_GaAs_, and the out-of-plane component is given by *ɛ*_⊥_=−(2*c*_12_/*c*_11_)*ɛ*_‖_, where *a*_AlGaAs_=(1−*x*) *a*_GaAs_+*xa*_AlAs_. The elastic constants *c*_11_ and *c*_12_ are taken as linear interpolations of those for GaAs and AlAs at 0 K. For GaAs, *c*_11_=11.26 and *c*_12_=5.71; for AlAs, *c*_11_=12.02 and *c*_12_=5.7 (ref. 26); all values have units of 10^11^ dyn cm^−2^. The energy levels of the heavy-hole and light-hole states can be expressed as follows according to the conventional perturbation treatment of the strain-induced valence-band splitting^32^:


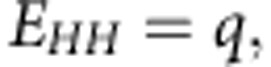



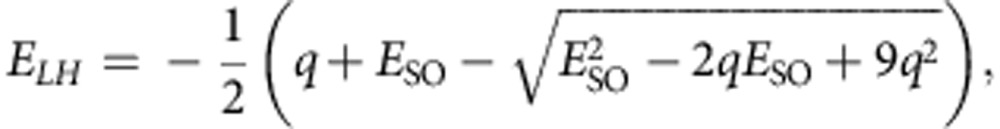


where *q*=*b*(*ɛ*_‖_−*ɛ*_⊥_) and *E*_SO_=(1−*x*)0.341 eV+*x*0.28 eV is the spin–orbit splitting in AlGaAs. The deformation potential *b* was taken to be −1.9 eV.

### Raman spectroscopy

A DCM dye laser and a Ti:sapphire laser were used as the excitation light sources for the excitation wavelength ranges of 640–695 nm and 700–910 nm, respectively. The excitation spot size was 1,000 × 100 μm^2^ for Al_*x*_Ga_1−*x*_As and 25 μm in diameter for GaAs_1−*x*_N_*x*_. Unless noted in the figures, all low-frequency spectra were collected in the ‘depolarized' configuration, where the laser light was linearly polarized, and the polarizing and analysing elements for the incoming and scattered light were arranged perpendicular to each other. The phonon spectrum in Fig. 1b was collected in the ‘polarized' configuration, where the polarizing and analysing elements for the incoming and scattered light were arranged in parallel. The Raman spectra were analysed using a charge-coupled device detector on a triple-stage Jobin Yvon T64000 spectrometer for the Al_*x*_Ga_1−*x*_As samples and on a single-stage spectrometer with a focal length of 0.27 m for the GaAs_1−*x*_N_*x*_ samples.

## Additional information

**How to cite this article:** Fluegel, B. *et al*. Electronic Raman scattering as an ultra-sensitive probe of strain effects in semiconductors. *Nat. Commun.* 6:7136 doi: 10.1038/ncomms8136 (2015).
